# The Book World of Medicine and Science

**Published:** 1900-10-20

**Authors:** 


### The Book World of Medicine and Science.

Operative and Practical Surgery for the Use of Students and Practitioners. By Thomas Carwardine, M.S. (Lond.), F.R.C.S, Assistant Surgeon Bristol Royal Infirmary. With 550 Illustrations. Bristol: John Wright and Co. 1900. Price 10s. 6d. net.

Some may perhaps wonder whether there are not books enough, and perhaps to spare, upon the art of surgery. But it is a progressive art, one which is constantly changing, and into which new methods and fresh devices are being introduced every day, and we must be grateful to those who at the expenditure of so much labour give to the profession the most recent information on the subject.

We need not say a word about the fitness of Mr. Carwardine, so far as opportunities and experience are concerned, for the task which he has undertaken, and no one who reads the book which has resulted from his labours can doubt that he has thrown his heart into the work, and has thus produced a handbook of the practical side of surgery which will be found of the greatest possible utility by students and practitioners.

The book appears to have been designed with the object of being a guide to clinical and practical surgical work rather than as a general handbook of surgery, and this must be borne in mind in considering its arrangement, for it will be noted that, although some subjects, such as fractures, are fully dealt with, others, such as certain more advanced operations which would only be likely to be performed by surgeons of some standing, are entered into with less completeness.

After a short chapter on surgical bacteriology, contributed by Dr. J. O. Symes, Mr. Carwardine gives a good description of the various bandages, splints, and like appliances, and this is followed by a couple of useful chapters, the one on dislocations and the other on fractures. In the latter most of the ordinary fractures are separately dealt with, and the appliances required in their treatment are discussed.

The section on operations is full of practical hints. Instruments, sutures, ligatures, incisions, the arrangement of the operating-room, and the various manipulations which are required in the different operations mentioned as well as in the after-dressing of the cases, are all well described and profusely illustrated. After this come separate chapters on the head, the neck and chest, genito-urinary surgery, the abdominal cavity, the mid-gut, the hind-gut, the teeth, nose, larynx, ear, and eye. In these latter chapters the author has had the assistance of various specialists, Dr. Watson Williams having contributed those on the examination of the nose and larynx, Mr. W. R. Acland on the extraction of teeth, Mr. W. H. Harsant on diseases of the ear, and Mr. F. R. Cross on the examination and treatment of the eye. The whole book will be found full of interesting matter, and will, we think, not only prove an admirable guide to students and house surgeons in their daily theatre and ward work, but will also find a special place in the hands of the general practitioner, acting, as it must do, as a means of mental refreshment in regard to the many details about which it is so easy to become rusty. We may add that if the said practitioner has been half-a-dozen years away from hospital work, this book will also certainly tell him a good deal that will be entirely new.

It is admirably printed, and the illustrations, if sometimes a little formal, capitally fulfil their purpose, serving well to render plain the author's meaning.

The Bacteriology of Everyday Practice. By J. O. Symes, M.D. Pp. 90. Crown 8vo. London: Baillière, Tindall, and Cox. 1900. Price 2s. 6d. net.

In the language of the advertisements this little book may be said to supply a long-felt want. In spite of the enormous bulk of recent bacteriological literature there is, so far as we are aware, no volume of merit equal to this, supplying in a concise form such bacteriological information as may be of practical value to the busy practitioner. Within the small compass of this little book we have an accurate account of those cases in which bacteriological methods assist clinical diagnosis, a description of the means of securing and identifying such microscopic specimens as may be readily prepared, and clear directions for taking cultures and preserving tissues for transmission to a laboratory. The information that is given is accurate, and no superfluous matter is inserted. The language employed is clear and precise; the statements sufficiently positive. The only suggestion that we feel inclined to make is that the value of the volume would be considerably enhanced by a few clear and bold illustrations.

Electricity in Gynæcology. By R. J. Cowen, L.R.C.S.I., L.R.C.P.I., &c. Pp. 132. (London: Baillière, Tindall, and Cox. 1900. Price 3s. 6d.)

In this *brochure* Mr. R. J. Cowen, whose name, as far as we can ascertain, has not hitherto been connected with gynæcological literature, seeks to direct the general practitioner in the use of electricity in gynæcology. We learn from the preface that only those methods which have been proved of service are described in the text. We are glad of it; but should have been still better pleased if Mr. Cowen had relieved the descriptions of his ingenious manipulations by some details of his successful results. Mr. Cowen draws sparks from the lumbar region in "primary amenorrhœa;" practises with a bipolar intra-uterine electrode in young married women of deficient sexual feeling; faradises the vagina at the menopause; galvanises pelvic exudations, prolapsed uteri, anteflexions, and retroversions. Nor does Mr. Cowen consider electricity of less value in subinvolution, endometritis, endo-salpingitis, ovaritis, pyo-salpingitis, hydro-salpingitis, fibroid tumours, and extra-uterine gestation. The various procedures are well and clearly described; the accounts and woodcuts of the various instruments employed leave nothing to be desired.

### BOOKS RECEIVED.

Baillière, Tindall and Cox.

